# A Timing Effect of 17-β Estradiol on Atherosclerotic Lesion Development in Female ApoE^−/−^ Mice

**DOI:** 10.3390/ijms21134710

**Published:** 2020-07-01

**Authors:** Obialunanma V. Ebenebe, Zoe Ashley, Jeffrey R. Erickson, Alison K. Heather

**Affiliations:** 1 Department of Physiology and Heart Otago, School of Biomedical Sciences, University of Otago, Dunedin 9054, New Zealand; oebeneb1@jhmi.edu (O.V.E.); zoe.ashley@otago.ac.nz (Z.A.); jeff.erickson@otago.ac.nz (J.R.E.); 2 Kohr Laboratory of Cardiovascular Redox Signaling, Bloomberg School of Public Health, Johns Hopkins University, Baltimore, MD 21205, USA

**Keywords:** atherosclerosis, calcification, fibrosis, intimal thickening, menopause, estrogen therapy

## Abstract

Differences in size or composition of existing plaques at the initiation of estrogen (E2) therapy may underpin evidence of increased risk of atherosclerosis-associated clinical sequelae. We investigated whether E2 had divergent effects on actively-growing versus established-advanced atherosclerotic lesions. Eight weeks of subcutaneous bi-weekly injections of 3 µg/g 17β-estradiol (*n* = 18) or vehicle control (*n* = 22) were administered to female Apolipoprotein null-mice aged 25- or 45 weeks old. Histological assessment of lesion size within the brachiocephalic artery was conducted. Lesion composition was also assessed with acellular, calcification and fibrosis areas measured and other cellular features (intimal thickening, foam cells, lipid pools and cholesterol) scored (0–3) for severity. The comparison showed increased lesion size and calcified area with advancing age but no effect of E2. However, subtle changes in composition were observed following E2. Within the younger group, E2 increased intima thickening and acceleration of calcification. In the older group, E2 increased the thickness of the lesion cap. Therefore, this study shows different effects of E2 depending on the underlying stage of lesion development at the time of initiation of treatment. These divergent changes help explain the controversy of the adverse effects of E2 treatment in cardiovascular disease.

## 1. Introduction

Epidemiology studies reveal that younger females (<50 years) have a lower atherosclerotic burden compared to males of the same age, suggesting that endogenous estradiol is cardioprotective [1,2]. This estradiol protective hypothesis is supported by increased cardiovascular disease risk following menopause [3]. Estradiol (or estrogen) therapy to counter the decline in endogenous levels post-menopause has been shown to reap beneficial cardiovascular effects (EPAT and ELITE trials) [4,5]. However, such treatment remains controversial, as estradiol therapy in post-menopausal females failed to prove beneficial in a number of randomised clinical trials (WELL-HART and KEEPS trials) [6,7]. Moreover, estradiol therapy has been associated with increased strokes [8]. The fundamental reasons for these apparent divergent effects are not clear but could be associated with differences in the underlying level of atherosclerotic disease.

Arterial calcification correlates with increased risk of clinical events, such as myocardial infarction and stroke [9]. Thus, increased calcification may be connected with the detrimental effects of estradiol therapy. Importantly, whether 17-β estradiol (E2) has effects on calcification remains under debate. Estrogen has been shown to be an independent predictor of atherosclerotic plaque calcification [10], and reduced E2 levels are associated with increased calcification [11,12,13]. Clinically, however, evidence of a positive correlation of E2 and calcification has been demonstrated [14], and in vitro E2 treatment induces calcification of cultured vascular smooth muscle cells (VSMC) [15] through the promotion of differentiation into osteoblast-like cells [16]. In contrast, E2 has been shown to reduce calcification [17] and maintain VSMCs in a contractile phenotype [18].

This controversy of effect of E2 on calcification also exists in the apolipoprotein E-null (ApoE^−/−^) mouse model of atherosclerosis. Increases [16], decreases [19] or no change [16] in the size of the calcified plaque area have been observed following E2 treatment. The divergent effects of E2 may be linked to the timing of treatment, with a “critical window” for treatment; beneficial effects observed if treatment is initiated early before lesions are established, but not when treatment is started in the presence of established advanced lesions [5,16]. Our previous study showed divergent effects of E2 within the same gonadally intact female ApoE^−/−^ mice, with augmentation of calcification in lesions that were established and growing, but no effect on established advanced lesions [16]. It was unclear from this previous study if the conflicting observations were due to timing and underlying atherosclerotic stage [20] or arterial site-specific effects [21], with different effects of E2 on the aortic sinus and brachiocephalic artery (BCA). Therefore, our present study aimed to interrogate only the timing hypothesis by concentrating on a single arterial site, the BCA, and assessing the effect of initiating E2 treatment at either 25- or 45 weeks of age, where lesions were actively-growing or established-advanced, respectively.

## 2. Results

### 2.1. E2 Treatment Does Not Affect Physiological Parameters

The animal growth profile over the treatment period was not altered by E2, with bodyweight gains of 2.0 ± 0.8 g compared to 3.4 ± 0.7 g (*p >* 0.05, *n* = 8–12) in the 25-week for vehicle and E2 groups, respectively. Similarly, in the 45-week group, no difference in body weight occurred with treatment, with gains of 0.1 ± 0.8 g compared to 0.8 ± 0.9 g (*p >* 0.05, *n* = 10). Serum E2 and lipid profiles were measured in our previous study [16], which showed elevated E2 and unchanged total cholesterol and high density lipid levels with a similar E2 dose.

### 2.2. Atherosclerotic Lesion Burden Is Not Enhanced by E2

**Figure 1:**
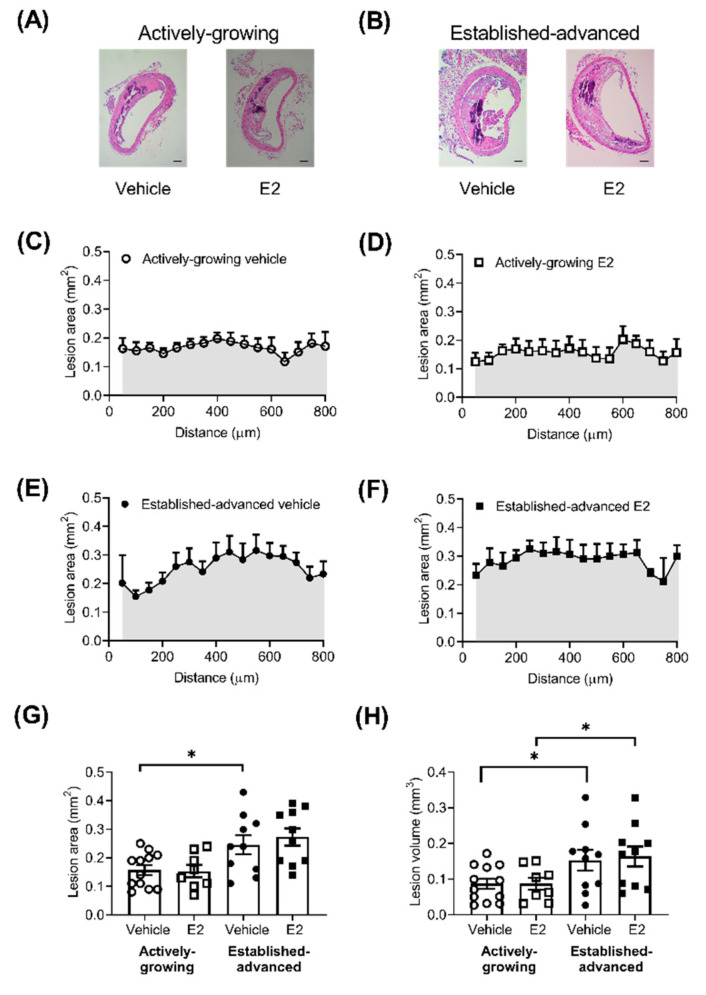
No effect of E2 on lesion size in actively-growing and established-advanced lesions. Atherosclerotic lesion burden was assessed by measurement of the lesion area, along the length of the brachiocephalic artery (BCA). Representative images of BCA from (**A**) actively-growing and (**B**) established-advanced H&E stained sections from either vehicle or E2 treated animals (scale bar = 100 µm). Lesion area (mm^2^) along the length of the BCA in the vehicle (circles) and E2 (squares) treated group for (**C**,**D**) growing (open symbols) and (**E**,**F**) advanced (closed symbols) lesions. (**G**) Average lesion area (mm^2^) and (**H**) area under the curve of lesion area and distance (shaded areas) indicates lesion volume (mm^3^) in the BCA with progression between actively-growing and established-advanced lesions but no effect of E2. Data is mean ± SEM, with *n* = 8–12. Mixed-effects two-way or one-way ANOVA, with Tukey’s post-tests, were used to determine statistical differences with * *p <* 0.05 difference between actively-growing and established-advanced.

Atherosclerotic lesions were observed in all BCA examined, and lesions were generally located on the lateral wall of the BCA (Figure 1A,B). The lesion area showed some variation along the length of the BCA (Figure 1C–F), but any differences observed were not significant. Along the length of the BCA, lesion size increased (*p <* 0.0001) between growing (25-weeks) and advanced (45-weeks) groups, which translated to a significant increase in the average lesion area (Figure 1G). Treatment of mice with E2 did not alter the lesion area (Figure 1G) in either the growing (Figure 1C,D) or advanced lesions (Figure 1E,F). The area under the curve of lesion area (mm^2^) along the length of the BCA provides an estimation of the total volume of the lesion within the BCA, which was significantly increased by age but not E2 treatment (Figure 1H). There was also no difference in the proportion of intimal surface involved as a result of E2 treatment with a difference of 6 ± 4 and 0 ± 3 % (*p >* 0.05) of the intimal surface affected in actively-growing and established-advanced, respectively.

### 2.3. Composition of Growing Lesions is Affected by E2

**Figure 2:**
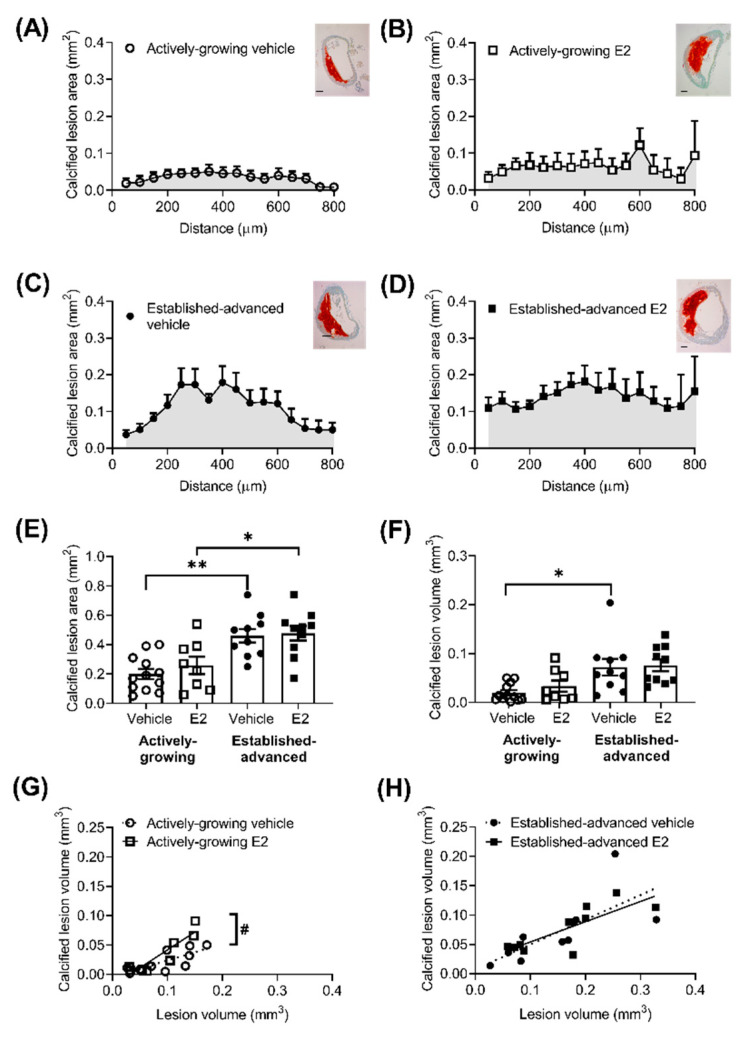
Increased calcification of growing atherosclerotic lesions after E2 treatment. Calcified area (mm^2^) of atherosclerotic lesions was measured from Alizarin Red stained sections of the brachiocephalic artery (BCA), with representative images shown (scale bars = 100 µm). Area of red stain along the length BCA in (**A**,**B**) actively-growing (open symbols) and (**C**,**D**) established-advanced lesions (closed symbols) for the vehicle (circles) and E2 (squares) treated groups and the average calcified lesion (**E**) area was calculated. The area under the curve provides an estimate of calcified volume (**F**), which positively correlated with lesion volume in both actively-growing (**G**) and established-advanced (**H**) lesions. Data is mean ± SEM, with *n* = 8–12. Mixed-effects two-way or one-way ANOVA, with Tukey’s post-test was used to determine statistical differences between groups and linear regression used to determine the relationship between calcified volume and lesion volume. Significance between actively-growing and established-advanced is depicted by * *p* < 0.05 and ** *p <* 0.01, whereas difference between vehicle and E2 are shown as # *p <* 0.05.

Lesion composition is an important factor when considering the effect of E2 on the progression of atherosclerotic lesions, particularly the amount of calcification, fibrosis and necrotic areas within the lesion. The extent of calcified lesion area (mm^2^) was measured along the length of the BCA (Figure 2). As expected, there was a clear age effect in calcified deposits, with more pronounced Alizarin Red staining in established-advanced relative to actively-growing lesions (Figure 2A–D). This translated to significant increases in the calcified area (Figure 2E) and calcified volume (Figure 2F) with increased age, although E2 treatment had no significant effect. However, we observed a shift in the distribution of the calcified deposits as a consequence of E2 treatment in the established-advanced lesions. Figure 2C shows that in vehicle-treated lesions larger calcified deposits were centered in the middle and smaller calcified areas closer to the ends of the BCA. In the E2-treated mice, the size of calcified deposits within the lesions were similar along the length of the artery (Figure 2D). Despite this apparent variation, the maximum calcification was observed at similar positions along the BCA (450 ± 91 and 385 ± 76 µm, *p* = 0.87 from the origin, in-vehicle and E2, respectively). The calcified volume positively correlated with lesion volume in both growing (*R*^2^ = 0.63, *p* = 0.002) (Figure 2G) and advanced (*R*^2^ = 0.52, *p =* 0.019) (Figure 2H) lesions. Notably, E2 treatment increased this correlation in the actively-growing lesions (*p* = 0.02) (Figure 2G), but this effect was not maintained in the established-advanced lesions. Therefore, our data show a modest acceleration of calcification in actively-growing lesions; in that, for similar-sized lesions, the amount of calcification was elevated following E2 treatment.

**Figure 3:**
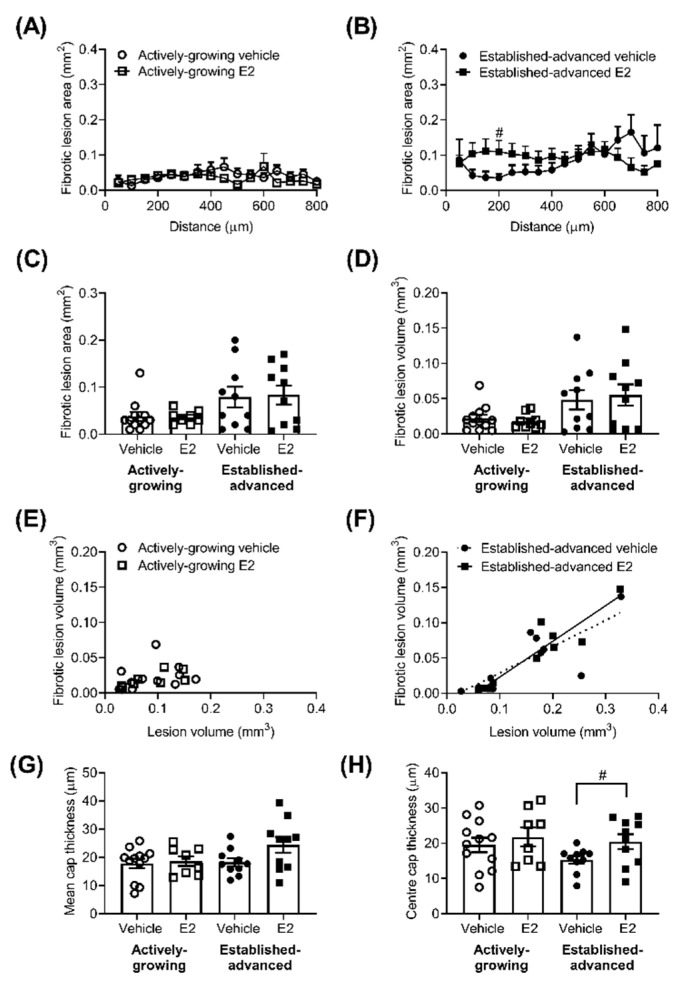
The fibrotic area of the atherosclerotic lesion is unaltered by E2. The fibrotic area of atherosclerotic lesions was measured along the length of the brachiocephalic artery (BCA). Fibrotic area (mm^2^) in the vehicle (circles) and E2 (squares) treated group for actively-growing (**A**) and established-advanced lesions (**B**), and the average fibrotic area (mm^2^) calculated (**C**). The area under the curve provides fibrotic volume (mm^3^) (**D**), which positively correlated with lesion volume in established-advanced (**F**) but not actively-growing (**E**) groups. The average thickness of the lesion cap (**G**) showed no effect of E2 or age, although the thickness of the cap at the mid-lateral position showed E2 prevention of thinning (**H**). Data is mean ± SEM, with *n* = 8–12. Mixed-effects repeated measures or Brown–Forsythe ANOVA was used to determine statistical differences as appropriate and linear regression was used to determine the relationship between fibrotic volume and lesion volume. Significance between vehicle and E2 is shown as # *p* < 0.05.

The extent of fibrosis along the length of the BCA showed a generalised increase in fibrosis (*p <* 0.01) as lesions progressed from actively-growing to established-advanced (Figure 3A,B). In the established-advanced lesions, E2 altered the profile along the length of the artery, with an increase in the fibrotic area closer to the origin at the aortic arch, statistically significant at 200 µm along with the BCA.

However, these changes did not fully translate into increases in the average fibrotic area (Figure 3C) or volume (Figure 3D). Fibrosis volume positively correlated with lesion volume (Figure 3D) in the advanced lesions (*R*^2^ = 0.63, *p* = 0.007 and *R*^2^ = 0.87, *p* < 0.0001, for vehicle and E2, respectively) but, interestingly, not in developing (Figure 3C) lesions (*R*^2^ = 0.09, *p* = 0.33 and *R*^2^ = 0.46, *p* = 0.06, for vehicle and E2, respectively). The average thickness of the lesion cap was measured and showed no significant effect of age or E2 (Figure 3E). Interestingly, the cap at the centre of the lesion (i.e., mid-lateral position) showed a trend for thinning as a result of increased age (*p* = 0.11) in vehicle-treated animals (Figure 3F). This age-related reduction was prevented by E2 treatment, with significantly (*p* = 0.04) thicker lesion cap in established advanced E2 treated lesions compared to vehicle-treated (Figure 3F).

**Figure 4:**
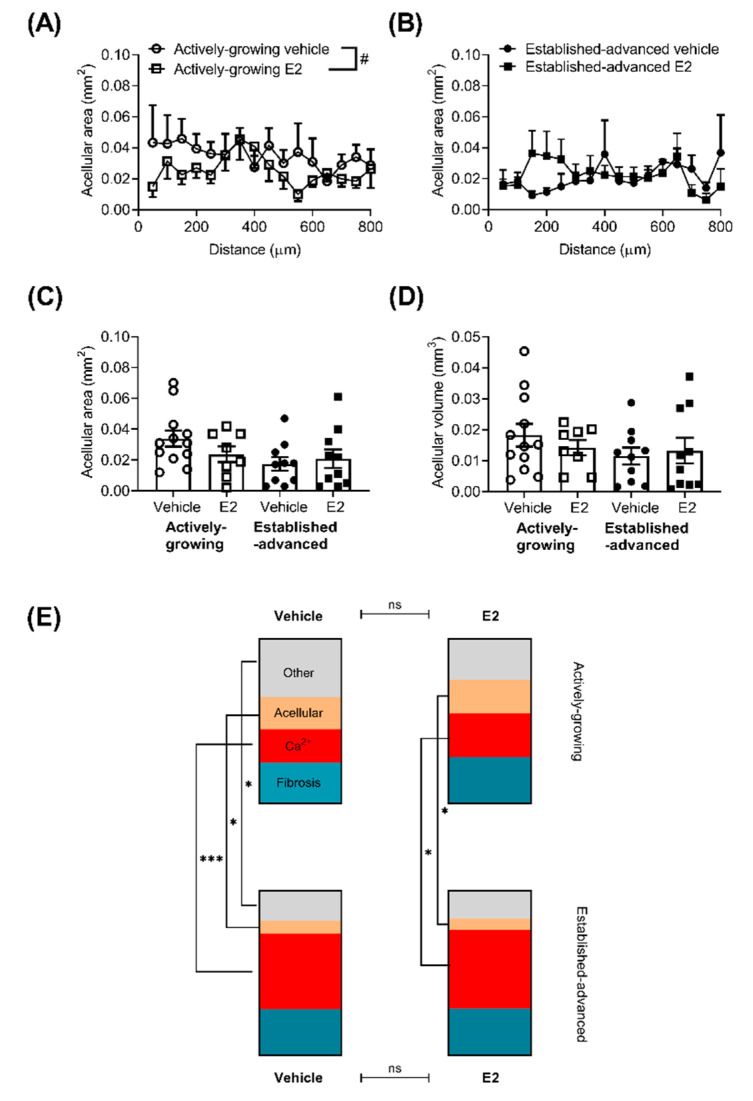
Minimal effect of E2 on the overall composition of atherosclerotic lesions. The acellular area, defined as areas devoid of nuclei, but excluding calcified tissue, was measured along the length of the brachiocephalic artery (BCA) for actively-growing (**A**) and established-advanced (**B**), with E2 inducing a small decrease in the acellular core area. The average acellular area (**C**) and volume (**D**) was not significantly different. The overall composition of atherosclerotic lesions within the BCA in terms of percentage of the lesion (**E**) that was calcified (red), fibrotic (teal), acellular (beige) or other cellular features (grey) was calculated for all four groups. The make-up of the lesions altered between actively-growing and established-advanced, indicative of lesion progression, but this was unaffected by E2 treatment. Data shows mean for *n* = 8–12. Mixed-effects two-way repeated measures or one-way ANOVA was used to determine statistical differences in the acellular area over the length of the BCA. A comparison of each cellular feature was assessed separately with ANOVA with Holm–Sidak post-tests. Significant differences between actively-growing and established-advanced are shown as * *p* < 0.05, *** *p* < 0.001, and differences between E2 and vehicle shown by # *p* < 0.05.

Another key component in atherosclerotic lesions in the necrotic core or acellular area within the lesion (Figure 4). In this case, the acellular area was defined as areas that were negative for nuclei on H&E stained sections, but excluded areas that demonstrated calcification. The acellular area along the length of the BCA varied in both actively growing (Figure 4A) and established-advanced (Figure 4B).

In actively-growing lesions, there was an overall significant difference between vehicle and E2 treatment (*p <* 0.01), although post-hoc testing was unable to determine any differences at specific positions along the length of the BCA. No differences in the average acellular area (Figure 4C) or acellular volume (Figure 4D) were observed between any group.

As lesions progress the size increases (Figure 1), but also the overall composition in terms of the proportion of lesion that is acellular, calcified, fibrotic or other components is altered. Calculation of the percentage of the lesion of the different components demonstrated this lesion progression, with a greater contribution of calcium and fibrotic deposits to the overall lesion in the advanced, compared to developing lesions, alongside decreases in the acellular area (Figure 4E). However, E2 did not alter the overall composition of the lesions in either actively-growing or established-advanced lesions.

**Figure 5:**
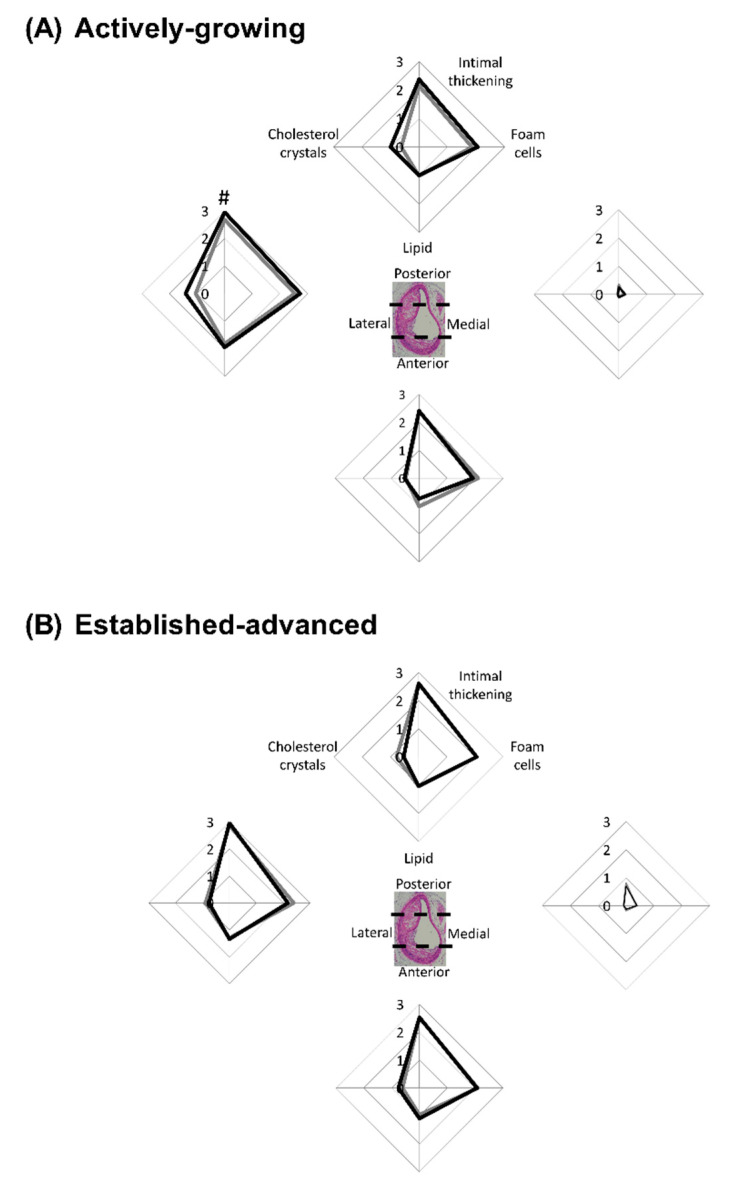
E2 increases atherosclerotic cellular feature scores in actively-growing lesions. Atherosclerotic lesions were scored based on the presence of key cellular features. Lesions were scored from 0 (not present) to 3 (substantial presence) in terms of intimal thickening, foam cells, lipid pools and cholesterol crystals. Each cellular feature was scored in each of the 4 regions of the artery for (**A**) actively-growing and (**B**) established-advanced lesions for the vehicle (grey-lines) and E2 (black-lines) treated animals. Data shows mean for *n* = 8–12. A comparison of the vehicle to E2 for each cellular feature was assessed separately with unpaired *t*-test with significance set at *p* < 0.05, with differences between vehicle and E2 indicated by #.

In addition to the acellular, calcified and fibrotic aspects, the cellular make-up of the lesions is also important. Key cellular features such as intimal thickening, foam cell presence, lipid deposition, and cholesterol crystal formation were scored to indicate the extent of severity (Figure 5).

As shown previously for this mouse model, lesions developed in an eccentric manner along the entire length of the BCA [16,22], and each region was assessed separately. The medial-region of the BCA, relative to the other regions, was protected from cellular changes (Figure 5A,B) in both age groups, although there were some pockets of intimal thickening, as demonstrated by the increased score, observed in the 45-week group (Figure 5B). This suggests lesion expansion into this area of the arterial wall as the disease progresses.

In the other circumferential regions of the BCA intimal thickening, lipid and cholesterol crystal deposition were present. In striking contrast to the medial-region, the lateral-region was most severely affected across both age groups (Figure 5A,B). This analysis provides insight into the growth of plaque in the BCA and most likely represents that the lateral wall of the BCA is exposed to altered shear stress forces and other influences of atherosclerotic progression [23].

With regard to E2 treatment, data showed no significant difference in the cellular composition of lesions between the vehicle and E2-treated mice in both age groups. However, in considering intimal thickening alone there was a significant (*p* = 0.04) increase in the lateral-region of the BCA in E2-treated mice within the younger group, relative to vehicle control (Figure 5A). The difference induced by E2 was not maintained through to the 45-week old mice, where intimal thickening had increased extensively in both groups, concomitant with age-induced plaque growth. This finding suggests that as a lesion is growing, E2 can induce small, but significant, effects, such as intimal thickening. Yet, as lesions progress, these effects are deemed insignificant in the face of the other strong driving factors such as the vicious inflammatory and oxidative stress processes that primarily drive lesion growth.

### 2.4. Clinical Based Assessment of Atherosclerotic Lesions

The extent of stenosis is an important determinant of clinical consequences and was estimated using a visual guide [24]. There was no difference in the average amount of stenosis of the BCA between the older and younger groups, nor an effect of E2 treatment (Table 1). Similarly, there were no differences observed in the maximum stenosis or location along the length of the BCA where maximum stenosis was detected. Hence, data shows that E2 treatment, whether started when lesions are established but growing versus established and advanced, does not augment remodelling to further restrict blood flow through the BCA.

**Figure 6:**
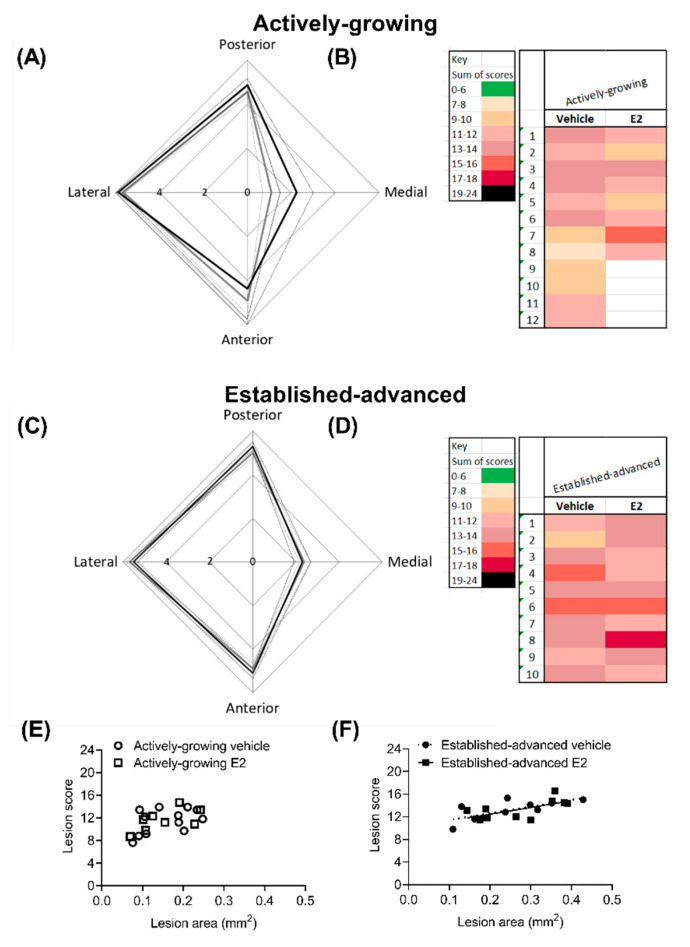
Overall lesion severity is not altered by E2 treatment. Lesion severity was scored with a modified clinical scoring system, with each circumferential area (posterior, medial, anterior and lateral) of the BCA scored separately. The maximal score shows the eccentric development of lesions in both (**A**) actively-growing and (**C**) established-advanced lesions, for both vehicle (grey-lines) and E2 (black-lines). The cumulative score from each of the 4 regions provided a heat map to indicate overall lesion severity for individual animals. The cumulative score positively correlated with lesion volume in established-advanced (**F**) but not actively-growing (**E**) lesions. Data is shown as (**A**,**C**) mean ± SEM (dotted-line) and (**B**,**D**) individual animals, with *n* = 8–12. Differences between vehicle and E2 were determined with unpaired *t*-test and linear regression was used to determine the relationship between lesion score and lesion area, with significance set at *p <* 0.05.

A modified clinical scoring system (Table A1) was used to indicate lesion severity with values from 0 (normal) to 6 (severe) assigned to each region of the BCA (Figure 6A,C) [25]. This scoring system again highlighted the lateral-region of the BCA as the most affected with a score of 6 (severe) in both established-advanced and actively-growing lesions. When considering the effect of E2-treatment there was no statistical difference between vehicle and E2, thereby highlighting that E2 treatment did not overtly alter the severity of the lesion.

The clinical score from each of the regions was summated for individual animals, with a maximal score of 24 indicative of severe lesion that encompasses the entire circumference of the BCA (Figure 6B,D). In all cases, no lesions had a summated score that indicated mild (green) or very severe (black) scores. The combined score was indicative of progression in severity between growing and advanced lesions, with darker hues observed on the advanced heat map (Figure 6C). However, in both growing and advanced groups, there was no difference (*p* = 0.12) between the average cumulative score as a result of E2 treatment (Table 2). Lesion score showed a positive correlation with lesion area in the established-advanced (*R*^2^ = 0.497, *p* = 0.02 and *R*^2^ = 0.416, *p* = 0.04 for vehicle and E2, respectively) and the regression lines between vehicle and E2 were not different (*p* = 0.99). This correlation between score and lesion area was not observed in actively-growing lesions, indicative of the less developed nature of these lesions.

## 3. Discussion

The major finding of this study is that E2 promotes subtle but significant changes to the cellular composition of actively-growing lesions and established-advanced lesions in the BCA, but the effects differ depending on the timing of the E2 treatment. These compositional changes are unlikely to be associated with altered clinical sequelae, and thus cannot fully explain the controversy of E2 treatment. Future studies should investigate if similar changes occur at other key arterial sites, as this could provide enlightenment on the increased cardiovascular disease risk.

Traditionally, there was thought to be an association between the size of lesions and the associated degree of stenosis and the risk of cardiovascular consequences [26]. Atherosclerotic lesions within the BCA increased in size and severity score with advancing age. This progression was expected due to the natural development of lesions and was similar to published data [20,27,28]. The progression was, however, unaffected by E2, which supports our earlier findings in 34-week old mice [16]. Therefore, independent of the stage of lesion development, actively-growing (25 weeks), established (34 weeks, [16]) or established-advanced (45 weeks), E2 does not drive changes to lesion size or overall severity.

Recently, plaque characteristics other than size have been identified to potentially influence the functional consequences of atherosclerosis, including the eccentricity of the lesion [29]. Our data is suggestive of eccentric expansion of lesions following E2 treatment with a subtle increase in lesion severity score in the protected medial-region in the actively-growing lesions, to a level that was similar to that observed in the established-advanced (Figure 6). This increase in score arose due to the presence of small pockets of intimal thickening and isolated foam cells at the margins of the growing atherosclerotic lesions. This is indicative of the acceleration of lesion progression by E2 in actively-growing atherosclerotic lesions.

In addition to lesion size, arterial calcification is indicative of disease severity [30] and is associated with an increased risk of adverse cardiovascular events [9,31,32]. Calcified area and volume were increased in the established-advanced compared to the actively-growing lesions. This increase with age is in line with previous studies [28,33] and further demonstrates lesion progression with advancing age. E2 treatment did not alter the average calcification area or volume. However, E2 did induce a subtle increase along the length of the BCA alongside an increase in the correlation between lesion and calcification volume in the actively-growing, but not fully established-advanced, lesions. The presence of larger calcified areas following E2 treatment in lesions of similar size indicates an increase in severity and acceleration of lesion progression. Overall, our data suggest a divergent effect of E2 based on timing with the acceleration of calcification in actively-growing lesions. A higher prevalence of intraplaque hemorrhage and calcification in stroke patients indicates an association between increased calcification and plaque vulnerability [34]. Therefore, the acceleration of calcification in the actively-growing lesions by E2 thereby could potentially increase the risk of ischemic events.

The distribution of calcium within atherosclerotic lesions is also important in determining the susceptibility of the plaque to rupture and ensuing clinical consequences [34]. In advanced-lesions, calcification distribution was altered from a centralized deposition in the control-treated group to a more expansive even distribution along the length of the BCA in the E2-treated group, although without a change in overall calcified area or volume.

Another process of atherosclerosis that has a direct role in plaque vulnerability is the development and subsequent thinning of the lesion cap [35]. This study was not designed to examine plaque rupture characteristics specifically, although E2 did influence the thickness of the lesion cap. In keeping with the fact that additional manipulations, such as high-fat diet, are required to drive atherosclerotic lesions in ApoE^−/−^ mice to rupture [36] our data showed minimal thinning (loss of 4 µm) of the cap as lesions progressed from actively-growing to established-advanced. Interestingly, at the most affected region of the artery, (i.e., the mid-lateral position) E2 treatment prevented this thinning and the cap was significantly thicker than in vehicle-treated arteries. This suggests that E2 could provide a protective effect against plaque rupture on established-advanced lesions, and warrants further investigations in a rupture-prone model to confirm this beneficial effect.

Our results show that the effect of E2 treatment is dependent on when treatment is initiated; with small but significant changes in plaque composition such as intimal thickening and calcification observed if treatment was started when atherosclerotic plaques were still actively-growing, which could lead to more clinical events. Initiation of E2 treatment when plaques were already fully formed and at an advanced stage, any subtle effect of E2 was muted by the dominating atherosclerotic processes most-likely mediated by well-described oxidative and inflammatory pathways (reviewed in [29]). However, E2 protected the lesion cap from thinning in these more advanced lesions, which could be protective. Thereby our data provide supportive evidence for the timing hypothesis of E2 treatment.

We know from the literature that if E2 is started before plaques are established, then E2 is highly protective [37,38,39]. We also know that endogenous E2 is highly protective in the ApoE^−/−^ mouse model as ovariectomy increases atherosclerosis in young mice [39,40,41]. Thus, relating this information to the clinical arena, it is possible that E2 therapy would be most beneficial to those women starting therapy when their arteries do not show overt atherosclerosis. Women that have established-advanced atherosclerosis at the time of E2 therapy may benefit through stabilization of lesions, and women that have actively-growing atherosclerotic lesions may even be at risk from an adverse effect of E2 treatment due to the subtle changes in composition (e.g., acceleration of calcification) of the atherosclerotic lesions. Our data suggest that female patients should be screened for the presence and severity of atherosclerotic lesions prior to receiving E2 therapy.

Female mice enter senescence rather than menopause, around the age of 40–45 weeks [42]. The standard approach to study menopause effects in mice is to perform ovariectomy. However, this leads to other effects including increased plasma cholesterol [41] which further accelerates atherosclerosis in this ApoE^−/−^ mouse model. We used intact mice to study the influence of E2 alone on atherosclerotic plaques, without the confounding lipid effects. However, this could be a limitation of our study as the effect of E2 on the calcification of vascular smooth muscle cells is influenced by the underlying hormonal status of the donor [18]. In future experiments, the use of hormonal regulated menopause, to reduce background E2 should be considered [43]. Another limitation of the study was that lesion development in the ApoE^−/−^ mice is dramatic and driven by increased circulating LDL levels, which our previous study shows is not further elevated by E2 treatment [16]. In humans, there is no such dominating generic pro-atherogenic background and therefore this model may not perfectly reflect the human clinical situation [44]. Hence, in the absence of such a strong driver of atherosclerotic disease, these subtle adverse E2 effects may have more significance.

In summary, we have investigated the timing hypothesis that exogenous E2 can modulate atherosclerotic lesion formation and composition depending on the stage and severity of underlying atherosclerotic disease at the start of treatment. We have shown that, in pre-existing but still developing atherosclerotic lesions, exogenous E2 can subtly promote expansion and calcification of the lesions, whereas in more established lesions E2 has a protective effect. These subtle changes in composition in actively-growing lesions are indicative of the acceleration of the disease process and could go some way to explain the controversy of adverse effects of E2 treatment in cardiovascular disease.

## 4. Materials and Methods

### 4.1. Animals

All procedures were approved by the University of Otago Animal Ethics Committee (AEC D74/14, 8 October 2014) and conducted in accordance with the New Zealand Animal Welfare Act. Forty female adult *ApoE*^−/−^ mice (strain name: B6.129P2-Apoetm1Unc/JArc) were used from an in-house colony derived from an Animal Resources Centre founder colony (Perth, Australia). Animals were housed in groups within small ventilated cages (GM500 Techniplast, Sydney, Australia) with standard environmental enrichment provided on a 12 h light/dark cycle at 21 ± 0.2 °C and 65 ± 2% relative humidity. Mice were maintained throughout on standard mouse chow (Teklad Global 18% Protein Rodent diet, Envigo, Madison, WI, USA) and water ad libitum.

The study used an unpaired design, conducted in a block fashion. Mice were randomised to either vehicle or 17-β estradiol (E2) treatment groups, with 8 weeks of bi-weekly subcutaneous injections of vehicle (10% ethanol in corn oil) or E2 (3 µg/g, Calbiochem, Sigma-Aldrich, St Louis, MO, USA). The treatment was initiated at either 25 or 45 (± 1) weeks of age. At these ages, *ApoE*^−/−^ mice are known to have actively growing and established-advanced lesions, respectively [22,23].

At the end of the treatment, mice were euthanised with CO_2_, followed by exsanguination. The mice were perfused and fixed with 4% paraformaldehyde pH 7.3 (Merck Millipore, Burlington, MA, USA) before the brachiocephalic artery (BCA) was removed from its origin at the aortic arch to the bifurcation into the right carotid and subclavian arteries. The arteries underwent a further 24 h of fixation in 4% paraformaldehyde at room temperature. Arteries were processed and embedded in paraffin wax, with the consistent orientation of artery to enable accurate identification of medial, lateral, posterior and anterior portions on sections of the BCA.

### 4.2. Histopathological Processing

Transverse 4 µm sections were prepared along the entire length of the BCA to provide an overall indication of the total lesion burden within the artery. At 50 µm intervals, sequential sections were stained with haematoxylin and eosin (H&E) to identify general cellular features, picrosirius red to identify fibrotic tissue and Alizarin Red to identify calcified deposits [45]. Digital images of stained sections were collected (Moticam 580 digital camera attached to Miotic BA310 light microscope, Xiamen, China) at ×10 magnification. Histological assessments were performed with the assessor blinded to the treatment group.

### 4.3. Lesion Assessment

#### 4.3.1. Lesion Measurements

**Figure A1:**
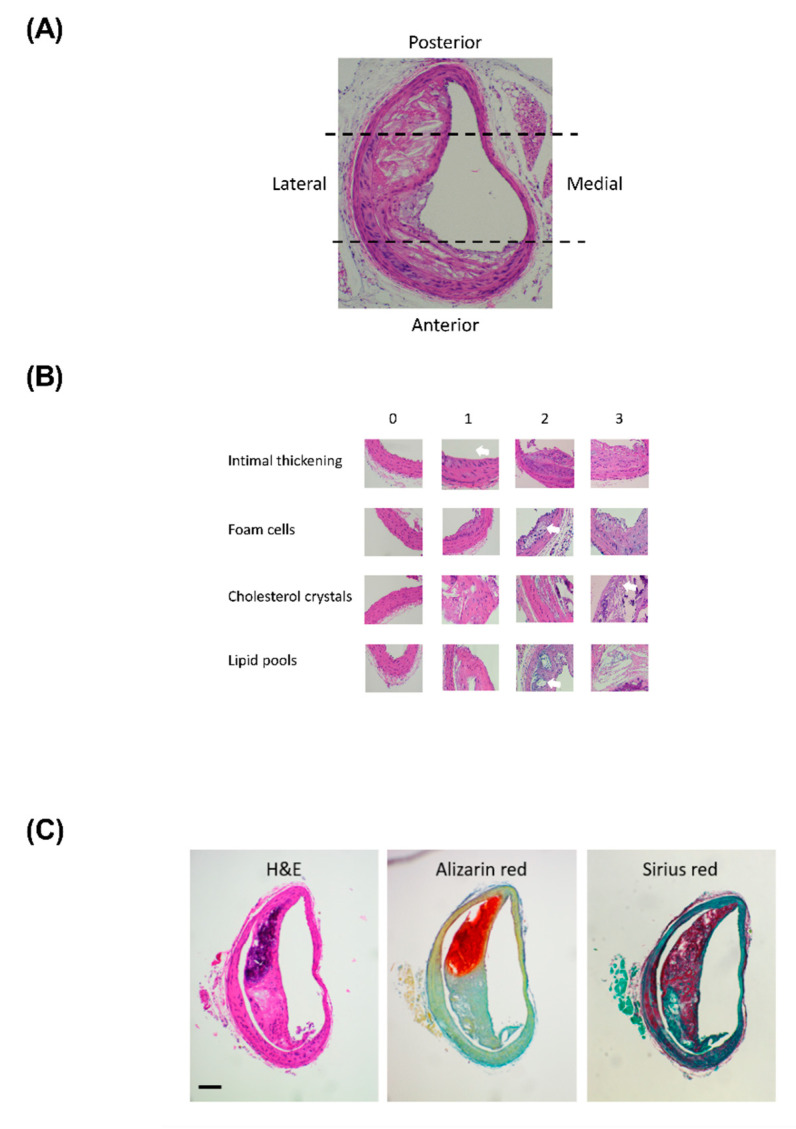
Cellular scoring example images. Representative image showing (**A**) the orientation of the BCA sections and the virtual division into the 4 regions and (**B**) representative images illustrating the differences between the scores (0–3) for the cellular features. Cellular features are identified by white arrows. One limitation of this histological preparation is the removal of lipids during the preparation and staining of paraffin-embedded sections; therefore, lipids were defined as empty holes. (**C**) Representative serial sections stained images demonstrating confirmation of fibrosis with picrosirius red stain, scale bar represents 100 µm.

The total lesion area (mm^2^), area of calcified deposits (red) and area of fibrotic tissue (blue) was measured on sections along the length of the BCA (Image J FIJI, [46]). Fibrotic area measurements were validated by comparison to measurements on picrosirus red-stained sections in a small subset of sections (Appendix Figure A1). The area under the curve of lesion, calcification and fibrosis (mm^2^) areas along the length of the BCA were calculated to provide total volume (mm^3^) within the BCA. The necrotic core area was measured on H&E sections, with the core defined as the area that was devoid of nuclei, including areas of cholesterol and lipid pools, but excluding areas of calcification. The overall makeup of the atherosclerotic lesions was calculated as a percentage of the lesion, in terms of necrotic core, calcification, fibrosis, with the remaining area designated as other components.

#### 4.3.2. Lesion Cellular Composition

Lesions develop asymmetrically within the BCA (Figure 1A,B), with lesions concentrated on the lateral aspect of the BCA. Therefore, the BCA was virtually divided into 4 regions: posterior, anterior, medial and lateral; with each region assessed separately. The cellular composition of the lesions was assessed on H&E sections in terms of intimal thickening, presence of foam cells, lipid pools and cholesterol crystals. Each feature was scored separately from 0 (none) through to 3 (substantial) linked to anchor images (Appendix Figure A1)

Luminal stenosis was estimated from H&E sections, based on published morphometrically generated visual guides [26]. This method was validated in a small subset of random sections by comparison to measured stenosis value; with no significant difference (*p* = 0.27) between the stenosis percentage obtained by the two methods (45.6 ± 7 vs 48.4 ± 7% for visual and measured, respectively).

To provide an overall indication of lesion severity, images were scored using a modified clinical scoring system [27] based on key cellular features from 0–6 (Appendix Table A1), with each anatomical region of the BCA scored separately. The sum of the scores from the four regions was calculated to indicate overall lesion severity score; with a maximal score of 24 indicative of severe lesion encompassing the entire circumference of the BCA.

#### 4.3.3. Data Analysis

All statistical analyses were performed using Prism v. 8.0.2. (Graphpad, San Diego, CA, USA). Data are presented as mean ± SEM, with n in parentheses. Differences between all the groups were determined with one-way ANOVA with Tukey’s multiple comparison tests or Brown–Forsythe ANOVA if standard deviations were deemed unequal or mixed-effects model two-way ANOVA with Holm–Sidak’s multiple comparison post-tests as appropriate. The effect of E2 on cellular scoring was determined by unpaired Student’s *t*-test in each of the four regions. The relationship between fibrosis or calcification and overall lesion area was tested with linear regression, with an assessment on whether the slopes were equally used to determine differences between vehicle and E2. Statistical significance level was set at *p* < 0.05, with * highlighting differences between actively-growing and established-advanced lesions and # used to display differences between vehicle and E2 treatment.

## Figures and Tables

**Table 1:** Stenosis along the length of the BCA was visually scored using a published guide [24], with the average and maximum stenosis levels reported, alongside the position within the length of the BCA where maximal stenosis was observed. Data is mean ± SEM, with *n* = 8–12, with no differences observed, as determined by ANOVA (*p >* 0.05).

Stenosis Measures	Actively-Growing		Established Advanced	
	Vehicle	E2	Vehicle	E2
Average stenosis (%)	51 ± 5	54 ± 5	61 ± 3	66 ± 2
Maximum stenosis (%)	75 ± 4	83 ± 3	81 ± 3	79 ± 3
Location of max stenosis (µm)	400 ± 56	513 ± 65	575 ± 93	375 ± 82

**Table 2:** The circumferential regions of the BCA were scored individually using a modified clinical score from 0 (normal) to 6 (severe lesion) and the scores summated to provide an overall atherosclerotic lesion severity score. A maximum score of 24 would indicate a severe lesion that encompasses the entire circumference of the BCA. Data is mean ± SEM, with *n* = 8–12, with no differences observed, as determined by ANOVA.

Group	Total Score
Actively-growing lesion vehicle	11 ± 0.6
Actively-growing lesion E2	12 ± 0.8
Established-advanced lesion vehicle	13 ± 0.6
Established-advanced lesion E2	13 ± 0.6

## Abbreviations

AEC	Animal Ethics Committee
ANOVA	Analysis of variance
ApoE^−/−^	apolipoprotein E-null mouse
BCA	Brachiocephalic artery
CO_2_	Carbon dioxide
E2	17β-estradiol
H&E	Hematoxylin and eosin
SEM	Standard error of mean
VSMC	Vascular smooth muscle cells

## Appendix A

**Table A1:** Modified clinical lesion score system for assessment of atherosclerotic lesion severity. The score is based on the type of lesion and the presence of key cellular features within the atherosclerotic lesion.

Score	Lesion Stage	Lesion Type	Key Cellular Features
0	No lesion	Normal	No abnormal features
1	Very early	Initial lesion	Minimal foam cells and intimal thickening
2	Early	Fatty streak	Increased intimal thickening and foam cells
3	Developing	Intermediate lesion	Extensive foam cell collection
4	Late-developing	Atheroma lesion	Lipid core and cholesterol crystals
5	Actively-growing	Fibroatheroma	Fibrosis and calcification
6	Established-advanced	Complicated lesion	Excessive calcification
